# ‘Flashforward’ mental imagery in adolescents: exploring developmental differences and associations with mental health

**DOI:** 10.1017/S1352465824000298

**Published:** 2024-09-23

**Authors:** A. Lau-Zhu, J. Stacey, D. Gibson, C. Chan, M. Cooper

**Affiliations:** 1 Department of Experimental Psychology, Medical Sciences Division, University of Oxford, Oxford, UK; 2 Division of Psychiatry, Department of Brain Sciences, Imperial College London, London, UK; 3 Child and Adolescent Mental Health Services, Oxford Health NHS Foundation Trust, Oxford, UK

**Keywords:** anxiety, episodic simulation, flashforward, future projection, future thinking, mental imagery

## Abstract

**Background:**

Future events can spring to mind unbidden in the form of involuntary mental images also known as ‘flashforwards’, which are deemed important for understanding and treating emotional distress. However, there has been little exploration of this form of imagery in youth, and even less so in those with high psychopathology vulnerabilities (e.g. due to developmental differences associated with neurodiversity or maltreatment).

**Aims:**

We aimed to test whether flashforwards are heightened (e.g. more frequent and emotional) in autistic and maltreatment-exposed adolescents relative to typically developing adolescents. We also explored their associations with anxiety/depression symptoms.

**Method:**

A survey including measures of flashforward imagery and mental health was completed by a group of adolescents (*n* = 87) aged 10–16 (and one of their caregivers) who met one of the following criteria: (i) had a diagnosis of autism spectrum disorder; (ii) a history of maltreatment; or (ii) no autism/maltreatment.

**Results:**

Flashforwards (i) were often of positive events and related to career, education and/or learning; with phenomenological properties (e.g. frequency and emotionality) that were (ii) not significantly different between groups; but nevertheless (iii) associated with symptoms of anxiety across groups (particularly for imagery emotionality), even after accounting for general trait (non-future) imagery vividness.

**Conclusions:**

As a modifiable cognitive risk factor, flashforward imagery warrants further consideration for understanding and improving mental health in young people. This implication may extend to range of developmental backgrounds, including autism and maltreatment.

## Introduction

Episodic future thinking, or episodic simulation, refers to the ability to project oneself into the future within specific scenarios, often recruiting mental imagery (Schacter *et al*., 2017). Functional accounts propose that this ability is crucial for a range of skills, including planning, problem solving and emotional regulation (Szpunar *et al*., 2014). While future thinking is thought to emerge early on in childhood (Steinberg *et al*., 2009), adolescence is arguably a key stage for its development. This period coincides with the critical life task of negotiating one’s past identity with future goals, in relation to education, career, relationships, and so forth (Johnson *et al*., 2014; Nurmi, 1991). Simultaneously, adolescence is also a period of increased emotionality and vulnerability when most mental health problems first emerge (Solmi *et al*., 2021). Taken together, there is a need to better understand the role of future thinking in adolescence and its link to psychopathology.

### Clinical and fundamental research on involuntary prospective imagery

Recent neuroscientific developments highlight the role of prospection in the simulation of threats (Bulley *et al*., 2017). A particular form of prospection, intrusive prospective imagery, is hypothesised to play an important role in the aetiology, maintenance and recovery of emotional disorders (Brewin *et al*., 2010). Such mental images, also known as ‘flashforwards’ (Engelhard *et al*., 2010; Hales *et al*., 2011), are involuntarily generated, perception-like, and emotionally charged. Flashforwards are common in anxiety disorders, where images frequently depict negative feared catastrophes. Examples include picturing oneself dying of cancer in health anxiety (e.g. Lau-Zhu and Brummer, 2022; Muse *et al*., 2010), being ridiculed by peers at an incoming party in social phobia (Chiu *et al*., 2022; Frets *et al*., 2014; Reimer and Moscovitch, 2015), or harming a loved one in obsessive compulsive disorder (e.g. Lau-Zhu *et al*., 2022; Rachman and de Silva, 1978). These flashforwards are thought to amplify threat appraisals and maladaptive coping (e.g. avoidance), maintaining anxiety problems (Brewin *et al*., 2010).

Involuntary mental images stand in contrast to those that are voluntarily generated (Lau-Zhu *et al*., 2019; Lau-Zhu *et al*., 2021; Pearson and Westbrook, 2015) and which have been traditionally neglected in mainstream cognitive research (Finnbogadóttir and Berntsen, 2013). However, there has been a recent surge in scientific interest on involuntary forms of future thinking in everyday life, which are often imagery-rich (Barsics *et al*., 2016; Cole and Kvavilashvili, 2019). Spontaneous thoughts in non-clinical populations are more often characterised by positive (rather than negative) future projections (Barsics *et al*., 2016; Finnbogadóttir and Berntsen, 2013). This positive bias may reflect a basic motivational tendency to seek out positive information to maintain a positive self-view, while distancing from threatening materials (Finnbogadóttir and Berntsen, 2013). The increased incidence or intensity of spontaneous positive imagery has been associated with higher levels of optimism (Beaty *et al*., 2019) and lower levels of depressive symptoms (Ji *et al*., 2019). Therefore, spontaneous future thinking arguably plays important functions including goal pursuits and emotional regulation (Barsics *et al*., 2016).

While experiencing spontaneous positive images can be beneficial (Barsics *et al*., 2016), in some cases they can also contribute to distress. Emotional dysregulation (in mood and anxiety disorders) has been associated with increased emotional impact of flashforward images of negative *and* positive events (Di Simplicio *et al*., 2016; Di Simplicio *et al*., 2019; van den Berg *et al*., 2020). Overly positive images can be found in bipolar mania (Ivins *et al*., 2014) and cravings in addictions (Kavanagh *et al*., 2005).

### Emerging research in youth populations

Intrusive emotional imagery research has so far been mostly restricted to adults (Brewin *et al*., 2010), even though adolescents appear to be more vivid imagers than adults (Gulyás *et al*., 2022). Studies with youth samples are, however, beginning to accumulate (Burnett Heyes *et al*., 2013; Lau-Zhu *et al*., 2022; Pile *et al*., 2021; Schwarz *et al*., 2020). The majority have focused on social anxiety (for a review, see Chapman *et al*., 2020), revealing that adolescents with social anxiety disorder report intrusive imagery of feared social scenarios, which maintains anxiety and contaminates social interactions (Leigh *et al*., 2020), although the time orientation of such imagery is not always queried.

In a recent study specifically querying for the future, greater impact of flashforwards of negative future events (e.g. ‘my dog getting ill’ or ‘my parents arguing’) was linked to more symptoms of generalised anxiety and depression in a community sample of adolescents (Pile and Lau, 2020). However, measurement of flashforwards here combined ratings of imagery with their associated responses (i.e. resulting arousal and avoidance), leaving it unclear whether and which aspect of flashforward imagery independently contributed to symptomatology. Flashforwards reported were also restricted to negative events, whereas recent work suggests that responses to both negative *and* positive future events (i.e. a general propensity to emotional imagery) can be pertinent to youth distress (Deeprose *et al*., 2011; O’Donnell *et al*., 2020).

Finally, prospective positive imagery has been investigated during daily life (Marciniak *et al*., 2023). This study, however, focused on deliberate generation (prompted by an app) rather than naturally occurring, involuntary generation central to the phenomenology of flashforwards.

### Developmental differences and psychopathology vulnerabilities

Existing research on involuntary mental images in youth has mainly focused on typically developing individuals (with anxiety problems). Our understanding of this form of cognition in those with developmental differences, which can heighten emotional vulnerabilities and often present to clinical services, remains limited. Here we consider autism spectrum and maltreatment history – two developmental presentations frequently overlooked in this line of research. We put forward three compelling reasons for considering these populations.

First, from an intervention standpoint, both autism and maltreatment are associated with elevated rates of mental health problems including anxiety and depression (Gilbert et al., 2009; Lai *et al*., 2019) but with reduced benefits from gold-standard psychological interventions (Lippard and Nemeroff, 2020; Weston *et al*., 2016). This clinical challenge suggests that imagery may represent a neglected target for improved interventions in these groups. Informed by existing cognitive accounts, one could predict that involuntary generation of emotional events (of both past and future orientation) is heightened in both autistic and maltreatment-exposed individuals relative to their typically developing peers, albeit through different mechanisms. In autism, reduced executive functioning could impair one’s ability to adaptively inhibit unwanted intrusions (McDonnell *et al*., 2017), leading to heightened flashforwards. In contrast, after maltreatment, excessive reliance on avoidance coping over time turns into a habitual cognitive style for managing threatening material, which paradoxically exacerbates intrusions (Hitchcock *et al*., 2017), including flashforwards.

Two studies hint at a potential heightened intrusion profile in these populations but have not probed underlying mechanisms. Ozsivadjian *et al*. (2017) found that autistic children reported more involuntary anxious images, but the future was not specifically queried. Steil *et al*. (2022) showed that maltreatment-exposed adolescents reported more negative involuntary images, but did not separate past and future events in their analyses.

Second, there is a pervasive assumption that autistic individuals are poor imagers (Dance *et al*., 2021) with limited imagination (Baron-Cohen *et al*., 2001), leading many clinicians to question the applicability of imagery-based interventions to improve autism mental health. While studies like that of Ozsivadjian *et al*. (2017) are beginning to challenge these assumptions, additional evidence is required.

Third, autism and maltreatment-related presentations have traditionally been studied separately, but there is a growing recognition of the need to understand their overlaps and distinctions (Dinkler *et al*., 2017; Gajwani and Minnis, 2023), especially in relation to cognitive profiles and processes (Lau-Zhu *et al*., 2024). This comparison is crucial because both conditions present with overlapping difficulties often complicating differential diagnosis and in turn the provision of appropriate support (Davidson *et al*., 2022; Moran, 2010).

### The present study

We sought to investigate flashforwards in adolescents aged 10–16, enriched with developmental differences, (i) to explore flashforward characteristics without constraining them for the first time, such as to a specific valence (Pile and Lau, 2020) or anxious scenarios only (Ozsivadjian *et al*., 2017). We then probed for (ii) differences in flashforwards between autistic adolescents, maltreatment-exposed adolescents, and adolescents without autism or maltreatment (typically developing adolescents for brevity); and (iii) associations between flashforward characteristics and symptoms of anxiety/depression; and (iv) associations between flashforward characteristics and executive functioning/avoidant coping.

As primary hypotheses, we predicted that (i) flashforward characteristics are heightened in autistic and maltreatment-exposed adolescents compared with typically developing adolescents and (ii) flashforward characteristics are associated with mental health (anxiety and depression).

As an exploratory hypothesis, we predicted that (iii) such flashforward characteristics are associated with avoidant coping (especially in the maltreatment-exposed group) and executive dysfunction (especially in the autism group).

## Method

### Participants

Eighty-seven UK-residing participants (aged 10–16) and their caregivers or parents took part. Adolescents aged below 16 provided online assent. Parents, caregivers and legal guardians and adolescents aged 16 provided online consent. For each adolescent, a small donation of £2 was given to a youth charity either working with the autism community (Parents Talking Asperger’s) or young people who had experienced significant early adversities (SAFE!). Data on different results from this same sample have been presented elsewhere (Lau-Zhu *et al*., 2024).

Recruitment sources included local child and adolescent mental health services, children’s social services, and community advertisement which included charities and university webpages. Adolescents in the autism group (*n* = 30) had a confirmed diagnosis of ASD based on the *Diagnostic and Statistical Manual* (DSM) 4th edition (American Psychiatric Association, 1994) or 5th edition (American Psychiatric Association; 2013), or the International Classification of Diseases (ICD) 10th edition (WHO; World Health Organization, 2016) or 11th edition (World Health Organization, 2019), by their keyworker (who had access to clinical records) or caregiver (who provided written evidence, if needed), and who also confirmed there was no indication of a history of maltreatment or prior social service contact due to the child’s quality of care. Adolescents in the maltreatment group (*n* = 28) had a documented history of maltreatment (i.e. sexual, physical or emotional abuse, and/or neglect) confirmed by their keyworkers, who also confirmed the absence of a (suspected) ASD diagnosis or first/second-degree relatives with ASD. Adolescents in the typically developing group (*n* = 29) had their caregivers confirm the absence of indicators (as described above) of either ASD or maltreatment history. Adolescents were not eligible if they: (1) did not read English; (2) had an organic brain condition (e.g. brain injury); (3) had experiences of psychotic episodes; (4) had a diagnosis of a learning disability or known IQ<70; (5) were actively suicidal; and/or (6) had sensory impediments that would interfere with completing questionnaires (e.g. difficulties with screen exposure >15 min).

### Measures

Adolescents and caregivers completed an online survey each (with self-reported measures) separately between July 2021 and April 2022.

#### Flashforwards survey

We integrated different approaches to assessing emotional imagery from previous work. Survey instructions and format were adapted for a study conducted during the COVID-19 pandemic in the UK, in consultation with autistic and maltreatment-exposed adolescents, their caregivers, and professionals working with them, to ensure understanding and feasibility while minimising burden.

First, we drew items from a youth version of the Impact of Future Event Scale (following Pile and Lau, 2020), which in turn was adapted from the original adult version (Deeprose and Holmes, 2010). Instruction began as follows: ‘The next questions are about how you think about the future. “Future” means anything that can happen tomorrow, or in the next few days, next few weeks, next few months, next few years, or even when you get much older. For each question, select how often it applies to you. The questions are about your experiences in the past 7 days’. Participants were then presented with four items of the intrusion subscale (e.g. ‘Do mental pictures about the future pop into your mind?’ and ‘Do you think about the future even when you don’t mean to?’), with four response options (‘not at all’, ‘rarely’, ‘sometimes’ and ‘often’). A total frequency score was the sum of all items, with values ranging from 0 to 20. This subscale showed good internal consistency in this study (Cronbach’s α = 0.73).

Second, participants were asked to provide a brief (the most important) example of what it is that they had been thinking might happen in the future for them. These responses were reviewed by the first author (A.L.-Z., who was blinded to group membership during coding) to categorise them based on previous research on spontaneous imagery in youth (Ozsivadjian *et al*., 2017; Pile and Lau, 2020). Twenty per cent of these were double-coded (by D.G.) with excellent agreement, Cohen’s kappa = 0.96 (Altman, 1991).

Third, participants answered four questions (following the Imagery Interview; Di Simplicio *et al*., 2016; Di Simplicio *et al*., 2019; Ozsivadjian *et al*., 2017) about additional phenomenological qualities of the reported significant image: (i) valence (‘Is this event positive or negative?’; response options were ‘positive’, ‘negative’ and ‘none’); (ii) vividness (‘How clear was this future event in your head when you imagined it?’); (iii) emotionality (‘How emotional did you feel when you imagined it?’); (iv) likelihood (‘How likely did you think this event was to happen?’). Response options (except for valence) used Likert scales of 1–5 anchored from ‘not at all’ to ‘extremely’.

Our assessment of flashforwards differed from prior work in two key ways. First, we did not include the arousal/avoidance subscales (Pile and Lau, 2020) as our main interest was in assessing imagery characteristics rather than its impact (and to reduce questionnaire length and construct overlap with measures of anxiety). Second, we did not constrain the intrusion scale to only negative events as we were interested in adolescents’ general propensity towards involuntary prospective imagery, which is more in line with the original conceptualisation in adults where both positive and negative events are included (Deeprose and Holmes, 2010). Finally, we considered a range of phenomenological characteristics beyond frequency of occurrence, in line with interview studies (Di Simplicio *et al*., 2016; Di Simplicio *et al*., 2019).

### Questionnaires

#### For adolescents

Anxiety and depression symptoms were assessed with the Revised Children’s Anxiety and Depression Scale-11 items (RCADS-11; Radez *et al*., 2021). PTSD symptoms were assessed with the Children’s Revised Impact of Event Scale (CRIES; Perrin *et al*., 2005), yielding separate subscales for re-experiencing and avoidance, the latter serving as an index of avoidant coping (Kuyken *et al*., 2006). A proxy for general cognitive ability (GCA) was performance using the Abbreviated 9-item form of the Raven’s Standard Progressive Matrices Test (RSPMT-9; Bilker *et al*., 2012), which is highly predictive of the original 60-item form (Raven, 2000) and recently used in adolescents (e.g. Bone *et al*., 2021; Morin *et al*., 2019). Trait (non-future) mental imagery vividness was assessed with the Plymouth Sensory Imagery Questionnaire, where adolescents rated vividness to imagined items across different sensory modalities (Andrade *et al*., 2013).

#### For caregivers

Caregivers completed questionnaires in relation to the child. Anxiety and depression symptoms were assessed with the RCADS-47 (Chorpita *et al*., 2005). Caregiver-rated PTSD symptoms based on *DSM-5* criteria (American Psychiatric Association, 2013) were assessed with the Child and Adolescent Trauma Screen (CATS; Sachser *et al*., 2017). Autistic traits were assessed with the Social Communication Questionnaire-Current version (SCQ; Rutter *et al*., 2003) with a cut-off score of ≥15 indicative of probable ASD (Chesnut *et al*., 2017). Everyday executive functioning was assessed with the Dysexecutive Questionnaire-Children (DEX-C; Emslie *et al*., 2003). Information on adolescents’ demographics, known diagnoses, and parental/caregiver highest educational level were also collected (the latter as a proxy for socioeconomic status or SES; Liberatos *et al*., 1988).

### Statistical analyses

Histograms were inspected to assess for normality. Homogeneity of variance was assessed using the Levene’s statistic. For continuous variables, overall differences across the three groups were assessed with one-way ANOVAs and follow-up independent-sample *t*-tests as appropriate. For categorical variables, group differences were assessed with chi-square tests. A two-tailed alpha level of .05 was used. Associations between flashforward characteristics and other variables (e.g. mental health) used Pearson’s correlation tests. Principal component analyses (varimax rotation and eigen value >1) were used to extract a common ‘depression’ and ‘anxiety’ components combining the adolescent and caregiver versions of the RCADS to generate a single score for each, therefore minimising the number of correlations performed. Sizes of correlations were compared with Fisher’s tests. Hierarchical multiple regressions were used to predict the influence of flashforward characteristics on anxiety and depression, after controlling for baseline variables that correlated with predictors and/or outcomes (i.e. age, sex, and trait imagery vividness). All statistical analyses were conducted in SPSS version 27 (IBM, 2020).

## Results

### Basic demographics and clinical profiles

There were no statistical differences between the three groups in terms of age, sex, SES and trait imagery vividness (across sensory modalities), although the proportion of adolescents described as of White ethnicity was highest in the autism group. Both the autism and maltreatment groups scored lowest on a caregiver-reported measure of executive functioning, while only the maltreatment group scored lowest on a measure of GCA. The number of autistic traits was highest in the autism group, followed by the maltreatment group and then the typically developing group. The number of past traumatic events was highest in the maltreatment group, followed by the autism group and then the typical group. Symptoms of emotional disorders were also highest in the autism and maltreatment groups. See Table 1.

To explore for potential covariates (see Table 2), across the whole sample, no significant correlations were found between clarity (vividness), emotionality and likelihood ratings of flashforwards and age, sex, ethnicity, SES or GCA. However, *frequency* of flashforwards significantly and positively correlated with age and sex, indicating that older and female adolescents reported higher occurrence of flashforwards. Trait imagery vividness was also positively correlated with *clarity* and *likelihood* ratings for flashfowards.

### Content of flashforwards

For valence, over half (56.3%) of flashforwards were endorsed as of ‘positive’ valence (20.7% as ‘negative’ and 23% reported as ‘none’) across groups. Thematically, most flashforwards across groups were about ‘career, education and/or learning’ (62.3%; e.g. ‘I may get a job as a scientist’; ‘moving to secondary school’; ‘I will be a dancer’) followed by ‘family, friends, and/or relationships’ (20.8%; e.g. ‘imagining my future wife’; ‘getting another sibling’; ‘living with my mum’). A small proportion was on other themes, including distress (6.5%; e.g. ‘anxious about what is going to happen in school’), leisure (6.5%; e.g. ‘getting really good at scootering’) and society (3.9%; e.g. ‘when cars are autopiloted’). The distributions of image types in terms of valence or themes did not significantly differ between groups, χ^2^’s<11.78, *p*>.162.

Finally, only a small proportion was thematically linked to concerns typical of *DSM*-5 anxiety disorders (26.6%; ‘having a bad reaction to COVID-19 and ending up in hospital’), *DSM*-5 traumatic events (7.6%; ‘dying traumatically’), or depicting interpersonal scenarios (36.7%; ‘people cancelling plans’).

### Phenomenological characteristics of flashforwards

Contrary to hypothesis, groups did not significantly differ on any flashforward characteristic considered (Table 3), including image *clarity, F*_2,84_ = 2.22, *p* = .115, η_p_^2^ = 0.05, *emotionality, F*_2,84_ = 2.06, *p* = .134, η_p_^2^ = 0.05, *likelihood, F*_2,84_ = 1.17, *p* = .317, η_p_^2^ = 0.03, and *frequency, F*_2,82_ = 1.12, *p* = .317, η_p_^2^ = 0.03. We repeated these analyses with positive images only (as these were the majority of images reported), but the same pattern of results remained. Excluding autistic participants with low SCQ scores (<15; Chesnut *et al*., 2017) or maltreatment-exposed participants with high SCQ scores (15 and above; Chesnut *et al*., 2017) did not change the pattern of findings.

### Associations with mental health

See Table 2 for correlational analyses. Partially consistent with hypotheses, flashforward *emotionality* was positively correlated with both anxiety and depression. Flashforward *frequency* was also positively correlated with both anxiety and depression. However, correlations between flashforward clarity/likelihood and anxiety/depression were not significant. When considering only adolescents who reported positive mental images, all the above correlations were no longer significant, except for a negative correlation between flashforward likelihood and depression, *r*(49) = –0.35, *p* = .013.

Hierarchical multiple regressions (see Table 4) were performed for anxiety and depression factors separately. In the first step, age, sex and trait vividness were entered, to control for these effects first. In the second step, flashforward emotionality and frequency (and also likelihood for depression factor only) were entered given the above significant correlations. We also explored moderation effects by groups in a third step, but these were not significant, so we omit them here for simplicity and due to their exploratory nature.

**Figure 1:**
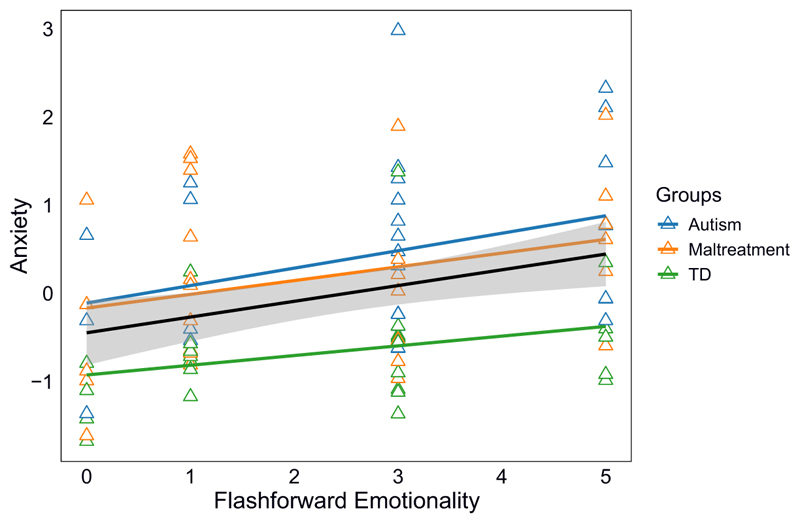
Association between Flashforward Emotionality and Anxiety, Overall and by Groups. *Note*. TD = typically developing; anxiety = factor score combining self- and caregiver RCADS scores; overall regression line includes 95% CI.

For the anxiety factor, the first model was significant, but no individual predictors were significant. The second model also including flashforward characteristics was also significant. Flashforward variables explained an additional 15% of variance. In this final adjusted model, flashforward emotionality was a significant *positive* predictor, meaning that the more emotional a self-relevant flashforward image was, the more anxiety symptoms were reported (Fig. 1). Trait imagery vividness was instead a significant *negative* predictor, meaning that better ability to generate vivid imagery of daily items was linked to less anxiety. Restricting the analyses to adolescents reporting positive images only (56.3% of the sample, *n* = 49), the models were no longer significant (likely owing to loss in power).

For the depression factor, the first model was statistically significant, with sex and age emerging as significant predictors. Female sex and older age predicted more depressive symptoms. The second model including flashforward characteristics was also significant. Flashforward variables explained an additional 7% of the variance. In this adjusted model, sex and age were no longer significant predictors. Instead, only trait imagery vividness was a significant *negative* predictor. That is, better ability to generate vivid imagery of daily items was linked to less depression. Restricting the analyses to adolescents reporting positive images only, both models remained significant but not any of the previous predictors (again likely owing to loss in power). However, flashforward *likelihood* emerged as a negative predictor, meaning that judging a positive flashforward as more likely to occur in real life was linked to less depression.

### Exploring candidate mechanisms

Across groups, avoidant coping (measured with the CRIES; see Table 1) was significantly and positively correlated with flashforward *emotionality, r*(84) = 0.30, *p* = .005, and *frequency, r*(84) = 0.36, *p*<.001, but not clarity/likelihood, *r*(84)<0.13, *p*>.251. Contrary to the hypothesis, these correlations were not significant when restricting the analyses to the maltreatment group only, *r*(28) = 0.05–0.31, *p*>.05.

Executive functioning (measured with the DEX-C; see Table 1) was not significantly correlated with any flashforward characteristics, *r*(84)<0.17, *p*>.332. However, when restricting analyses to the autism group only, and consistent with the hypothesis, worse executive functioning was correlated with increased flashforward *frequency* (controlling for sex and age), *r*(24) = –0.40, *p* = .045. This correlation in the autism group was significantly bigger than in the maltreatment group, *r*(22) = 0.14, *p* = .506 [*Z* = 1.78, *p* = .037], but not than in the typically developing group, *r*(25) = –0.40, *p* = .040 [*Z* = 1.02, *p* = .155].

## Discussion

We set out to explore involuntary *flashforward* imagery, and its relevance for mental health, in a sample of adolescents aged 10–16. Our preliminary work extends previous findings by considering a sample enriched with developmental differences with high psychopathology vulnerabilities, which partly reflected treatment-seeking young people within routine clinical services in the UK. Overall, we found links between flashforwards and anxiety which were applicable across the full sample, regardless of developmental backgrounds considered.

Unlike prior work looking at flashforwards in young people focusing on stressful events (e.g. Pile and Lau, 2020) or worries (Shukla *et al*., 2023), we did not restrict the valence of the mental image selected. Most adolescents selected a positive flashfowards image, consistent with emerging non-clinical studies (e.g. Barsics *et al*., 2016), and which may function to maintain a positive self-image (Finnbogadóttir and Berntsen, 2013). The most significant images were associated with career, education, and/or learning, which are key concerns of future orientation in adolescence (Nurmi, 1991; Sawyer *et al*., 2012) and in line with imagery’s role in representing goals (Barsics *et al*., 2016; Çili and Stopa, 2015). Those themes, albeit of mostly positive valence, are similar to the content of worries reported recently by young people during the COVID-19 pandemic (Shukla *et al*., 2023). In the absence of direct instructions to focus on the negative, positive images appear to be more salient for young people when thinking about the future.

Contrary to the heightened flashforward hypothesis, there were no significant group differences on phenomenological characteristics of flashforwards between autistic/maltreatment-exposed and typically developing adolescents. This finding is in opposition to the presence of significant group differences on another form of future thinking assessed in the same sample. We previously found that the specificity of voluntarily generated future events is reduced in maltreatment-exposed compared with typically developing adolescents (Lau-Zhu *et al*., 2024). Relatedly, we also did not find significant group differences in trait imagery vividness (of everyday scenes such as imagining certain sights and smells) in the current study (Andrade *et al*., 2013). Taken together, these add to the emerging picture that different aspects of emotional imagery dissociate, for instance between involuntary versus voluntary generation (Lau-Zhu *et al*., 2019; Lau-Zhu *et al*., 2021; Pearson and Westbrook, 2015) and subjective/emotional versus objective/cognitive aspects of imagery (Di Simplicio *et al*., 2016; Di Simplicio *et al*., 2019). It is still plausible that the heightened flashforward hypothesis is specific to negative rather than positive flashforwards, so measuring both separately would be an important next step. Future replications and extensions could also benefit from employing pre-registration, the lack of which is a limitation of our present study.

Flashforward *emotionality* emerged as the most consistent phenomenological characteristic predicting anxiety symptoms, after controlling for key covariates (including demographics and trait imagery vividness). Flashforward emotionality was not a significant predictor of depression, but this effect may become significant with a bigger sample. Nevertheless, the flashforward–anxiety association may still be bigger than a flashforward–depression one, which could be tested in future. With this caveat in mind, the current flashforward–anxiety association can be interpreted in several ways.

First, from a neuroscientific perspective, the evolved function of mental imagery is to predict threats (Schacter *et al*., 2017), suggesting its central role in the over-estimation of threats in clinical conditions. Indeed, intrusive flashforwards are prominent in anxiety disorders and a focus of treatment innovation (Brewin *et al*., 2010).

Second, considering that the emotionality rating captured both negative and positive images, it is plausible that imagery amplifies emotions of both valences, fuelling affect lability. For instance, in mania, positive images can become too intense and dysfunctional (Ivins *et al*., 2014). Affective lability, closely tied to anxiety (Di Simplicio *et al*., 2016; Di Simplicio *et al*., 2019; Lau-Zhu *et al*., 2023), is a common feature of autism (Mazefsky *et al*., 2013) and maltreatment (Dvir *et al*., 2014), both part of the developmental profiles of two-thirds of our sample (see Table 1). Thus, our flashforward measure may have tapped into a general propensity for experiencing *both* involuntary positive and negative imagery, as with the original conceptualisation in adults (Deeprose and Holmes, 2010).

Third, imagery’s appraisal may be important. A seemingly positive image (the majority in this sample) could have been appraised negatively. For example, an adolescent could hold a ‘positive’ image of becoming a scientist but then appraising it negatively as a difficult or uncertain goal. Imagery valence does not always match with appraisal valence (Ghita *et al*., 2021). Negative appraisals may have been more common given the COVID-19 context.

Measuring affect lability and appraisals could clarify the different possibilities above in future studies. Development of a youth-specific flashforward measure with these considerations could also be fruitful. Longitudinal designs using cohort research or experience sampling (Thunnissen *et al*., 2022) could provide converging evidence to our preliminary cross-sectional findings.

The absence of moderation effects by group implies that the emotion amplification effects of flashforwards are applicable to adolescents across various developmental differences. Our data, and recent work (Ozsivadjian *et al*., 2017), challenge long-held assumptions (often perpetuated in clinical settings) that all autistic individuals are poor imagers (Dance *et al*., 2021) or have limited imagination (Baron-Cohen *et al*., 2001). The inherent heterogeneity within autism (Happé and Frith, 2020) suggests that both weak and strong imagers can exist in the autism community. Individuals with mild-to-moderate intellectual disabilities (with or without autism) are also able to engage with mental imagery with appropriate support (Hewitt *et al*., 2022; Hewitt *et al*., 2023). Focusing on (flashforward) mental imagery, for whom it is relevant, could provide an important route towards therapeutic innovations to tackle the autism mental health challenge – a community priority (Weston *et al*., 2016).

Mechanistically, flashforward frequency appeared to be related to key candidate processes. Higher frequency was associated with more avoidant coping, in line with cognitive theory highlighting the counterproductive effects of avoidance on memory intrusions (Williams *et al*., 2007), and here extended to future-based intrusions. Higher flashforward frequency was also associated with less executive functioning, specifically in the autism group (O’Hearn *et al*., 2008), which can translate to detrimental effects over the control of flashforwards. Despite the absence of group differences in flashforward phenomenology (e.g. frequency or clarity), the underlying mechanisms may differ between groups. These analyses were exploratory but the obtained effect sizes pave the way for future mechanistic studies.

We also found that higher *likelihood* ratings on positive images were associated with fewer depressive symptoms, consistent with recent non-clinical studies (Beaty *et al*., 2019; Ji *et al*., 2019). An overly intense positive image (of a highly desired goal such as of future career) may lead to anxiety-linked dysregulation, but if it is underactive then it may confer risk for depression. It would be important to distinguish between the presence of unhelpful positive images from the absence of functional positive images, and to explore whether these factors distinctly influence mental health.

Clinically, enquiring about both negative and positive flashforwards more explicitly will be crucial in future, as these represent different intervention targets. Several imagery-based techniques have shown benefits in managing and/or reducing intrusive mental imagery in adults, such as imagery rescripting or metacognitive strategies aimed at changing the meaning or power of *dysfunctional* images, regardless of their valence (Hackmann *et al*., 2011; Lau-Zhu *et al*., 2023; Stopa, 2009). Recently, these techniques have been successfully applied in young people (Di Simplicio *et al*., 2020; Lau-Zhu *et al*., 2022; Pile *et al*., 2021). A particularly promising approach leverages simple, imagery-interfering tasks, which rely relatively less on language-based skills (Lau-Zhu *et al*., 2017; Lau-Zhu *et al*., 2019; Lau-Zhu *et al*., 2021; Lau-Zhu *et al*., 2022; Rackham and Lau-Zhu, 2021). Given the potential benefits on depression, *functional* forms of spontaneous positive images could also be promoted (Blackwell *et al*., 2020).

Our analyses revealed the presence of suppressor effects (McCurdy *et al*., 2023). Initially, trait imagery vividness (while controlling for age and sex) was *not* a significant predictor of anxiety/depression, but then became a significant *negative* predictor, when additional flashforward characteristics were controlled for (Table 4). This contrasts with a facet of flashforward imagery (i.e. its emotionality) as significant *positive* predictor of anxiety, demonstrating imagery dissociations. The trait imagery effect suggests the possibility that individuals who struggle with generating (and possibly flexibly manipulating) a variety of vivid mental scenes experience difficulties in downregulating affect lability, reflected in increased anxiety/depression symptoms. Overall, the relationship between different facets of imagery experience (across emotional and non-emotional domains) appears to be complex. Future investigations should consider these facets together rather than in isolation.

To conclude, the significance of future thinking in adolescent development is increasingly recognised, in particular its role in psychopathology. While there has been growing theoretical attention to involuntary mental imagery in emotional disorders, little exploration has been conducted in adolescents, especially among those with high psychopathology vulnerabilities due to developmental differences. Our preliminary work suggests that flashforward imagery holds relevance for understanding and improving mental health across adolescents with varying developmental profiles, including those related to autism and maltreatment. This approach is especially pertinent for anxiety problems, the incidence of which peaks in youth and where concerns about the future are central, often taking the form of imagery-rich cognitions. Further research is warranted to shed additional light on developmental, maintenance and intervention mechanisms.

## Figures and Tables

**Table 1:** Background variables including demographics and clinical measures by group

	Autism (*n* = 30)	Maltreatment (*n* = 28)	TH (*n* = 29)	Group comparisons (*p*-values)^d^
Age, years: mean (*SD*)	12.47 (1.98)	13.57 (2.08)	12.93 (1.98)	.118
Sex (at birth)^e^: females (%)	14 (47%)	17 (61%)	15 (52%)	.557
Ethnicity; White (%)	28 (93%)^a^	19 (68%)^b^	18 (62%)^b^	.013
Socioeconomic status (SES), parental education: *n* (%) at university level	21 (70%)	13 (54%)	19 (68%)	.437
General cognitive ability (GCA), RSPMT-9^h^: mean (*SD*)	4.85 (2.16)^a^	3.37 (1.50)^b^	5.29 (2.05)^a^	.001
Trait imagery vividness, Psi-Q: mean (*SD*)	6.46 (2.58)	5.27 (2.44)	5.58 (2.11)	.168
Diagnoses of neurodevelopmental disorders (others)^f^: *n* (%)	13 (43%)^a^	2 (7%)^b^	0^b^	<.001
Diagnoses of emotional disorders^g^: *n* (%)	8 (27%)^a^	2 (7%)^b^	0^b^	.004
Medication, yes: *n* (%)	14 (48%)^a^	1 (4%)^b^	0^b^	<.001
Talking therapy, yes: *n* (%)	13 (43%)^a^	18 (64%)^a^	0^b^	<.001
AS traits, SCQ^h^: mean (SD)	17.90 (5.72)^a^	10.77 (4.74)^b^	5.00 (3.45)^c^	<.001
Trauma history; lifetime n of DSM-5 traumatic events (CATS^h^): mdn	1^a^	3^b^	0^c^	<.001
Anxiety symptoms (self-report, RCADS-11^h^ total score: mean (*SD*)	7.66 (4.86)^a^	7.00 (5.18)^a^	4.64 (2.79)^b^	.030
Depression symptoms (self-report), RCADS-11^h^ total score: mean (*SD*)	5.69 (4.10)^a^	6.00 (4.24)^a^	3.75 (2.32)^b^	.050
Anxiety symptoms (caregiver report), RCADS-47^h^ t-scores: mean (*SD*)	74.34 (17.99)^a^	67.00 (15.84)^a^	49.03 (9.56)^b^	<.001
Depression symptoms (caregiver report), RCADS-47^h^ t-scores: mean (*SD*)	77.28 (18.14)^a^	68.39 (15.28)^a^	47.44 (6.97)^b^	<.001
Executive functioning, DEX-C^h^: mean (*SD*)	39.72 (15.08)^a^	33.81 (17.49)^a^	8.83 (7.50)^b^	.001
Avoidant coping, CRIES^h^ avoidant subscale; mean (*SD*)	9.96 (6.98)	9.71 (7.08)	6.68 (5.74)	.125

TD; typical development; RSPMT-9, Raven’s Standard Progressive Matrices Test 9-Items Short Form; DEX-C, Dysexecutive Questionnaire Child Version; Psi-Q, Plymouth Sensory Imagery Questionnaire; SCQ, Social Communication Questionnaire Current Version.

a,b,c Different letters are used to denote significant pairwise differences

d overall group comparisons (i.e. across the three groups)

e one participant in the autism group (female at birth) identified as transgender

f diagnoses of neurodevelopmental disorders included ADHD, Tourette’s syndrome, dyspraxia, dyscalculia, dyslexia, and sensory processing disorder

g diagnoses of emotional disorders included post-traumatic stress disorder, obsessive compulsive disorder, body dysmorphic disorder, depressive disorder, generalised anxiety disorder

h missing data were found for SES (maltreatment=4; TD=1); RSPMT-9 (autism=3, maltreatment=1, TD=1); Psy-Q (autism=3, maltreatment=1, TD=2); SCQ (maltreatment=2); RCADS-11 (autism=1; TD=1); DEX-C (autism: *n*=1, maltreatment: *n*=2); CRIES (autism=2; TD=1).

**Table 2:** Correlation matrix for key baseline variables, flashforward characteristics, and mental health, for the full sample

	1	2	3	4	5	6	7	8	9	10	11
1. Age	—										
2. Sex	0.23*	—									
3. Ethnicity	–0.07	0.09	—								
4. SES	0.02	0.04	–0.04	—							
5. GCA	0.14	–0.14	0.16	0.26*	—						
6. Trait imagery vividness	–0.16	–0.15	0.09	0.26*	0.14	—					
7. FF frequency	0.32**	0.24*	–0.03	0.09	0.19	0.17	—				
8. FF emotionality	0.07	0.17	0.14	0.15	–0.11	0.19	0.59***	—			
9. FF clarity	–0.20	–0.15	0.09	–0.04	0.17	0.39**	0.40***	0.37***	—		
10. FF likelihood	–0.02	–0.18	–0.04	0.08	0.16	0.22*	0.18	0.30**	0.46***		
11. Anxiety	0.15	0.28**	0.17	–0.04	–0.24*	–0.15	0.35***	0.42***	0.08	0.09	
12. Depression	0.31**	0.31**	0.17	–0.10	–0.14	–0.23*	0.35***	0.31**	0.03	–0.05	0.74***

SES, socioeconomic status, indexed by parental education (university *vs* non-university); GCA, general cognitive ability indexed by Raven’s Standard Progressive Matrices Test 9-Items Short Form; FF, flashforward; anxiety and depression scores were derived by combining self-report (RCADS-11) and caregiver-reports (RCADS-47) as a factor score; *n* per correlation varied from 82 to 87 due to missing data;

* *p*<.05,

** *p*<.01,

*** *p*<.001.

**Table 3:** Means and standard deviations for flashforward characteristics by group

	Autism (*n* = 30)	Maltreatment (*n* = 28)	TD (*n* = 29)
FF clarity (0–5)	3.54 (1.36)	2.86 (1.11)	2.86 (1.13)
FF emotionality (0–5)	2.06 (1.50)	2.79 (1.48)	2.68 (1.13)
FF likelihood (0–5)	3.27 (1.44)	2.82 (1.31)	3.31 (1.26)
FF frequency (0–20)	10.47 (4.43)	9.86 (5.88)	9.31 (4.71)

TD, typically developing; FF, flashforward.

**Table 4:** Two-step linear regression analyses with anxiety or depression scores as dependent variables

	Anxiety			Depression	
Independent variable	Model 1 β	Model 2 β		Model 1 β	Model 2 β
Age	0.09	0.02		0.25*	0.18
Sex	0.22	0.12		0.22*	0.12
Trait imagery vividness	–0.10	–0.22*		–0.16	–0.24*
Flashforward emotionality		0.36**			0.24
Flashforward frequency		0.12			0.15
Flashforward likelihood					–0.06
Adjusted *R^2^*	0.05	0.20		0.15	0.22
Δ*R^2^*	0.08	0.16		0.18	0.10
*F* for ΔR^2^	2.36*	8.26***		5.68**	3.46*
*F* for model	2.36*	4.99***		5.68**	4.84***

*n* = 82 (due to missing data on trait vividness; autism = 3, maltreatment = 1, typically developing = 2);

* *p*<.05,

** *p*<.01,

*** *p*<.001.

## Data Availability

The data that support the findings of this study are available from the corresponding author upon reasonable request.

## References

[R1] Altman DG (1991). Practical Statistics for Medical Research.

[R2] American Psychiatric Association (1994). Diagnostic and Statistical Manual of Mental Disorder.

[R3] American Psychiatric Association (2013). Diagnostic and Statistical Manual of Mental Disorder.

[R4] Andrade J, May J, Deeprose C, Baugh S, Ganis G (2013). Assessing Vividness of Mental Imagery: The Plymouth Sensory Imagery Questionnaire.

[R5] Baron-Cohen S, Wheelwright S, Skinner R, Martin J, Clubley E (2001). The Autism-Spectrum Quotient (AQ): evidence from Asperger syndrome/high-functioning autism, males and females, scientists and mathematicians. Journal of Autism and Developmental Disorders.

[R6] Barsics C, Van der Linden M, D’Argembeau A (2016). Frequency, characteristics, and perceived functions of emotional future thinking in daily life. Quaterly Journal of Experimental Psychology.

[R7] Beaty RE, Seli P, Schacter DL (2019). Thinking about the past and future in daily life: an experience sampling study of individual differences in mental time travel. Psychological Research.

[R8] Bilker WB, Hansen JA, Brensinger CM, Richard J, Gur RE, Gur RC (2012). Development of abbreviated nine-item forms of the Raven’s Standard Progressive Matrices Test. Assessment.

[R9] Blackwell SE, Dooley D, Würtz F, Woud ML, Margraf J (2020). Inducing positive involuntary mental imagery in everyday life: an experimental investigation. Memory.

[R10] Bone JK, Lewis G, Roiser JP, Blakemore SJ, Lewis G (2021). Recall bias during adolescence: gender differences and associations with depressive symptoms. Journal of Affective Disorders.

[R11] Brewin CR, Gregory JD, Lipton M, Burgess N (2010). Intrusive images in psychological disorders: characteristics, neural mechanisms, and treatment implications. Psychological Review.

[R12] Bulley A, Henry JD, Suddendorf T (2017). Thinking about threats: memory and prospection in human threat management. Consciousness and Cognition.

[R13] Burnett Heyes S, Lau JYF, Holmes EA (2013). Mental imagery, emotion and psychopathology across child and adolescent development. Developmental Cognitive Neuroscience.

[R14] Chapman J, Halldorsson B, Creswell C (2020). Mental imagery in social anxiety in children and young people: a systematic review. Clinical Child and Family Psychology Review.

[R15] Chesnut SR, Wei T, Barnard-Brak L, Richman DM (2017). A meta-analysis of the Social Communication Questionnaire: screening for autism spectrum disorder. Autism.

[R16] Chiu K, Clark DM, Leigh E (2022). Characterising negative mental imagery in adolescent social anxiety. Cognitive Therapy and Research.

[R17] Chorpita BF, Moffitt CE, Gray J (2005). Psychometric properties of the Revised Child Anxiety and Depression Scale in a clinical sample. Behaviour Research and Therapy.

[R18] Çili S, Stopa L (2015). Intrusive mental imagery in psychological disorders: Is the self the key to understanding maintenance?. Frontiers in Psychiatry.

[R19] Cole S, Kvavilashvili L (2019). Spontaneous future cognition: the past, present and future of an emerging topic. Psychological Research.

[R20] Dance CJ, Jaquiery M, Eagleman DM, Porteous D, Zeman A, Simner J (2021). What is the relationship between aphantasia, synaesthesia and autism?. Consciousness and Cognition.

[R21] Davidson C, Moran H, Minnis H (2022). Autism and attachment disorders – how do we tell the difference?. BJPsych Advances.

[R22] Deeprose C, Holmes EA (2010). An exploration of prospective imagery: the Impact of Future Events Scale. Behavioural and Cognitive Psychotherapy.

[R23] Deeprose C, Malik A, Holmes EA (2011). Measuring intrusive prospective imagery using the impact of future events scale: psychometric properties and relation to risk for bipolar disorder. International Journal of Cognitive Therapy.

[R24] Di Simplicio M, Appiah-Kusi E, Wilkinson P, Watson P, Meiser-Stedman C, Kavanagh DJ, Holmes EA (2020). Imaginator: a proof-of-concept feasibility trial of a brief imagery-based psychological intervention for young people who self-harm. Suicide and Life-Threatening Behavior.

[R25] Di Simplicio M, Lau-Zhu A, Meluken I, Taylor P, Kessing LV, Vinberg M, Holmes EA, Miskowiak KW (2019). Emotional mental imagery abnormalities in monozygotic twins with, at high-risk of, and without affective disorders: present in affected twins in remission but absent in high-risk twins. Frontiers in Psychiatry.

[R26] Di Simplicio M, Renner F, Blackwell SE, Mitchell H, Stratford HJ, Watson P, Myers N, Nobre AC, Lau-Zhu A, Holmes EA (2016). An investigation of mental imagery in bipolar disorder: exploring ‘the mind’s eye’. Bipolar Disorders.

[R27] Dinkler L, Lundström S, Gajwani R, Lichtenstein P, Gillberg C, Minnis H (2017). Maltreatment-associated neurodevelopmental disorders: a co-twin control analysis. Journal of Child Psychology and Psychiatry.

[R28] Dvir Y, Ford JD, Hill M, Frazier JA (2014). Childhood maltreatment, emotional dysregulation, and psychiatric comorbidities. Harvard Review of Psychiatry.

[R29] Emslie H, Wilson FC, Burden V, Nimmo-Smith I, Wilson BA (2003). Behavioural Assessment of the Dysexecutive Syndrome for Children (BADS-C).

[R30] Engelhard IM, Van den Hout MA, Janssen WC, van der Beek J (2010). Eye movements reduce vividness and emotionality of ‘flashforwards’. Behaviour Research and Therapy.

[R31] Finnbogadóttir H, Berntsen D (2013). Involuntary future projections are as frequent as involuntary memories, but more positive. Consciousness and Cognition.

[R32] Frets PG, Kevenaar C, Van Der Heiden C (2014). Imagery rescripting as a stand-alone treatment for patients with social phobia: a case series. Journal of Behavior Therapy and Experimental Psychiatry.

[R33] Gajwani R, Minnis H (2023). Double jeopardy: implications of neurodevelopmental conditions and adverse childhood experiences for child health. European Child & Adolescent Psychiatry.

[R34] Ghita A, Tooley E, Lawrence PJ (2021). Intrusive imagery in anxiety disorders in adolescents. Behavioural and Cognitive Psychotherapy.

[R35] Gilbert R, Widom CS, Browne K, Fergusson D, Webb E, Janson S (2009). Burden and consequences of child maltreatment in high-income countries. The Lancet.

[R36] Gulyás E, Gombos F, Sütöri S, Lovas A, Ziman G, Kovács I (2022). Visual imagery vividness declines across the lifespan. Cortex.

[R37] Hackmann A, Bennett-Levy J, Holmes Emily A (2011). Oxford Guide to Imagery in Cognitive Therapy.

[R38] Hales SA, Deeprose C, Goodwin GM, Holmes EA (2011). Cognitions in bipolar affective disorder and unipolar depression: imagining suicide. Bipolar Disorders.

[R39] Happé F, Frith U (2020). Annual Research Review: Looking back to look forward – changes in the concept of autism and implications for future research. Journal of Child Psychology and Psychiatry.

[R40] Hewitt OM, Langdon PE, Hales SA, Larkin M (2023). The phenomenology of mental imagery in people with intellectual disabilities. Psychology and Psychotherapy: Theory, Research and Practice.

[R41] Hewitt OM, Steel C, Hales SA, Hayden N, Gundeslioglu H, Tapp K, Langdon P (2022). A systematic review and narrative synthesis of mental imagery tasks in people with an intellectual disability: implications for psychological therapies. Clinical Psychology Review.

[R42] Hitchcock C, Werner-Seidler A, Blackwell SE, Dalgleish T (2017). Autobiographical episodic memory-based training for the treatment of mood, anxiety and stress-related disorders: a systematic review and meta-analysis. Clinical Psychology Review.

[R43] IBM (2020). IBM SPSS Statistics.

[R44] Ivins A, Di Simplicio M, Close H, Goodwin GM, Holmes E (2014). Mental imagery in bipolar affective disorder versus unipolar depression: investigating cognitions at times of ‘positive’ mood. Journal of Affective Disorders.

[R45] Ji JL, Holmes EA, MacLeod C, Murphy FC (2019). Spontaneous cognition in dysphoria: reduced positive bias in imagining the future. Psychological Research.

[R46] Johnson SRL, Blum RW, Cheng TL (2014). Future orientation: a construct with implications for adolescent health and wellbeing. International Journal of Adolescent Medicine and Health.

[R47] Kavanagh DJ, Andrade J, May J (2005). Imaginary relish and exquisite torture: the elaborated intrusion theory of desire. Psychological Review.

[R48] Kuyken W, Howell R, Dalgleish T (2006). Overgeneral autobiographical memory in depressed adolescents with, versus without, a reported history of trauma. Journal of Abnormal Psychology.

[R49] Lai MC, Kassee C, Besney R, Bonato S, Hull L, Mandy W, Szatmari P, Ameis SH (2019). Prevalence of co-occurring mental health diagnoses in the autism population: a systematic review and meta-analysis. The Lancet Psychiatry.

[R50] Lau-Zhu A, Brummer L (2022). A cognitive-behavioural approach to targeting sensation-based and intrusion-based misinterpretations in health anxiety: a single-case experimental study. Behaviour Change.

[R51] Lau-Zhu A, Chan C, Gibson D, Stark E, Wang J, Happé F, Stacey J, Cooper M (2024). Specificity of episodic future thinking in adolescents: comparing childhood maltreatment, autism spectrum and typical development. Research on Child and Adolescent Psychopathology.

[R52] Lau-Zhu A, Farrington A, Bissessar C (2022). Boosting exposure and response prevention with imagery-based techniques: a case study tackling sexual obsessions in an adolescent. the Cognitive Behaviour Therapist.

[R53] Lau-Zhu A, Henson RN, Holmes EA (2019). Intrusive memories and voluntary memory of a trauma film: effects of a cognitive interference task after encoding. Journal of Experimental Psychology: General.

[R54] Lau-Zhu A, Henson RN, Holmes EA (2021). Selectively interfering with intrusive but not voluntary memories of a trauma film: accounting for the role of associative memory. Clinical Psychological Science.

[R55] Lau-Zhu A, Holmes EA, Butterfield S, Holmes J (2017). Selective association between Tetris game play and visuospatial working memory: a preliminary investigation. Applied Cognitive Psychology.

[R56] Lau-Zhu A, Tuxen N, Roerne ML, Di Simplicio M (2023). Imagery-based cognitive therapy for comorbid anxiety in bipolar disorder: two case studies in Denmark. Psychiatry Research Case Reports.

[R57] Leigh E, Chiu K, Clark DM (2020). The effects of modifying mental imagery in adolescent social anxiety. PLoS One.

[R58] Liberatos P, Link BG, Kelsey JL (1988). The measurement of social class in epidemiology. Epidemiologic Reviews.

[R59] Lippard ETC, Nemeroff CB (2020). The devastating clinical consequences of child abuse and neglect: increased disease vulnerability and poor treatment response in mood disorders. American Journal of Psychiatry.

[R60] Marciniak MA, Shanahan L, Binder H, Kalisch R, Kleim B (2023). Positive prospective mental imagery characteristics in young adults and their associations with depressive symptoms. Cognitive Therapy and Research.

[R61] Mazefsky CA, Herrington J, Siegel M, Scarpa A, Maddox BB, Scahill L, White SW (2013). The role of emotion regulation in autism spectrum disorder: emotion regulation in ASD. Journal of the American Academy of Child and Adolescent Psychiatry.

[R62] McCurdy BH, Scozzafava MD, Bradley T, Matlow R, Weems CF, Carrion VG (2023). Impact of anxiety and depression on academic achievement among underserved school children: evidence of suppressor effects. Current Psychology.

[R63] McDonnell CG, Valentino K, Diehl JJ (2017). A developmental psychopathology perspective on autobiographical memory in autism spectrum disorder. Developmental Review.

[R64] Moran HJ (2010). Clinical observations of the differences between children in the autism spectrum and those with attachment problems: the Coventry Grid. Good Autism Practice.

[R65] Morin JFG, Afzali MH, Bourque J, Stewart SH, Séguin JR, O’Leary-Barrett M, Conrod PJ (2019). A population-based analysis of the relationship between substance use and adolescent cognitive development. American Journal of Psychiatry.

[R66] Muse K, McManus F, Hackmann A, Williams M, Williams M (2010). Intrusive imagery in severe health anxiety: prevalence, nature and links with memories and maintenance cycles. Behaviour Research and Therapy.

[R67] Nurmi JE (1991). How do adolescents see their future? A review of the development of future orientation and planning. Developmental Review.

[R68] O’Donnell C, Di Simplicio M, Burnett Heyes S (2020). Hypomanic-like experiences and spontaneous emotional mental imagery. Journal of Affective Disorders.

[R69] O’Hearn K, Asato M, Ordaz S, Luna B (2008). Neurodevelopment and executive function in autism. Development and Psychopathology.

[R70] Ozsivadjian A, Hollocks MJ, Southcott J, Absoud M, Holmes EA (2017). Anxious imagery in children with and without autism spectrum disorder: an investigation into occurrence, content, features and implications for therapy. Journal of Autism and Developmental Disorders.

[R71] Pearson J, Westbrook F (2015). Phantom perception: voluntary and involuntary nonretinal vision. Trends in Cognitive Sciences.

[R72] Perrin S, Meiser-Stedman R, Smith P (2005). The Children’s Revised Impact of Event Scale (CRIES): validity as a screening instrument for PTSD. Behavioural and Cognitive Psychotherapy.

[R73] Pile V, Lau JYF (2020). Intrusive images of a distressing future: links between prospective mental imagery, generalized anxiety and a tendency to suppress emotional experience in youth. Behaviour Research and Therapy.

[R74] Pile V, Williamson G, Saunders A, Holmes EA, Lau JYF (2021). Harnessing emotional mental imagery to reduce anxiety and depression in young people: an integrative review of progress and promise. The Lancet Psychiatry.

[R75] Rachman S, de Silva P (1978). Abnormal and normal obsessions. Behaviour Research and Therapy.

[R76] Rackham LA, Lau-Zhu A (2021). Taxing working memory to modulate mental imagery of the 9/11 terrorist attacks following media exposure during childhood: a pilot study in young adult UK residents. Anxiety, Stress and Coping.

[R77] Radez J, Waite P, Chorpita B, Creswell C, Orchard F, Percy R, Spence SH, Reardon T (2021). Using the 11-item version of the RCADS to identify anxiety and depressive disorders in adolescents. Research on Child and Adolescent Psychopathology.

[R78] Raven J (2000). The Raven’s Progressive Matrices: change and stability over culture and time. Cognitive Psychology.

[R79] Reimer SG, Moscovitch DA (2015). The impact of imagery rescripting on memory appraisals and core beliefs in social anxiety disorder. Behaviour Research and Therapy.

[R80] Rutter M, Bailey A, Lord C (2003). The Social Communication Questionnaire.

[R81] Sachser C, Berliner L, Holt T, Jensen TK, Jungbluth N, Risch E, Rosner R, Goldbeck L (2017). International development and psychometric properties of the Child and Adolescent Trauma Screen (CATS). Journal of Affective Disorders.

[R82] Sawyer SM, Afifi RA, Bearinger LH, Blakemore SJ, Dick B, Ezeh AC, Patton GC (2012). Adolescence: a foundation for future health. The Lancet.

[R83] Schacter DL, Benoit RG, Szpunar KK (2017). Episodic future thinking: mechanisms and functions. Current Opinion in Behavioral Sciences.

[R84] Schwarz S, Grasmann D, Schreiber F, Stangier U (2020). Mental imagery and its relevance for psychopathology and psychological treatment in children and adolescents: a systematic review. International Journal of Cognitive Therapy.

[R85] Shukla M, Crew A, Wu A, Riddleston L, Hutchinson T, Kumari V, Hughes LD, Lau JYF (2023). Self-reported worries in young people during the COVID-19 pandemic. Cognitive Therapy and Research.

[R86] Solmi M, Radua J, Olivola M, Croce E, Soardo L, Salazar de Pablo G, Shin J, Kirkbride JB, Jones P, Kim JH, Kim JY (2021). Age at onset of mental disorders worldwide: large-scale meta-analysis of 192 epidemiological studies. Molecular Psychiatry.

[R87] Steil R, Fischer A, Gutermann J, Rosner R (2022). Mental imagery in adolescent PTSD patients after child abuse: a comparison with matched healthy controls. BMC Psychiatry.

[R88] Steinberg L, Graham S, O’Brien L, Woolard J, Cauffman E, Banich M (2009). Age differences in future orientation and delay discounting. Child Development.

[R89] Stopa L (2009). Imagery and the Threatened Self: Perspectives on Mental Imagery and the Self in Cognitive Therapy.

[R90] Szpunar KK, Spreng RN, Schacter DL (2014). A taxonomy of prospection: Introducing an organizational framework for future-oriented cognition. Proceedings of the National Academy of Sciences of the USA.

[R91] Thunnissen MR, Aan het Rot M, van den Hoofdakker BJ, Nauta MH (2022). Youth psychopathology in daily life: systematically reviewed characteristics and potentials of ecological momentary assessment applications. Child Psychiatry and Human Development.

[R92] van den Berg KC, Voncken M, Hendrickson AT, Houterman S, Keijsers GPJ (2020). Image qualities and mood variability: evaluating the utility of an imagery survey for bipolar disorder. Journal of Affective Disorders.

[R93] Weston L, Hodgekins J, Langdon PE (2016). Effectiveness of cognitive behavioural therapy with people who have autistic spectrum disorders: a systematic review and meta-analysis. Clinical Psychology Review.

[R94] Williams JMG, Barnhofer T, Crane C, Hermans D, Raes F, Watkins E, Dalgleish T (2007). Autobiographical memory specificity and emotional disorder. Psychological Bulletin.

[R95] World Health Organization (2016). International Statistical Classification of Diseases and Related Health Problems.

[R96] World Health Organization (2019). International Statistical Classification of Diseases and Related Health Problems.

